# Concurrent Benign Metastasizing Leiomyoma in the Abdominal Wall and Pelvic Cavity: A Case Report and Review of the Literature

**DOI:** 10.3389/fsurg.2022.842707

**Published:** 2022-04-18

**Authors:** Yue Li, Tongtong Xu, Mingdan Wang, Lili Jiang, Qifang Liu, Kuiran Liu

**Affiliations:** Department of Gynecology and Obstetrics, Shengjing Hospital of China Medical University, Shenyang, China

**Keywords:** benign, metastasizing leiomyoma, pathological characteristics, diagnosis, differential diagnosis, treatment

## Abstract

Benign metastatic leiomyoma (BML) is a histologically benign disease with invasive biological behavior. Most patients are women of childbearing age with a history of uterine leiomyoma. The progress of the disease is relatively slow, the prognosis is good, and most patients can survive for a long time. The lung is the common metastatic site, and BML with metastatic lesions outside the lung is very rare. A 37-year-old woman with multiple BML in the abdominal wall and pelvic cavity after uterine leiomyoma surgery was admitted to our hospital. Combined with the clinical data of this case and reviewing the relevant literature, this paper discusses the pathological characteristics, diagnosis, differential diagnosis, and treatment of BML.

## Introduction

Benign metastatic leiomyoma (BML) is an extremely rare benign leiomyoma with invasive biological behavior. The disease was first proposed by Steiner (1), and most of the patients are women of childbearing age with a history of uterine leiomyoma surgery such as hysterectomy or myomectomy (2). The most common site of BML is the lung. It can also occur in the right atrium, pelvic and abdominal cavity, omentum, lymph nodes, muscle tissue, brain, bone, and in the abdominal wall skin and scars (3–5). Herein, we introduce a rare case with multiple BML in abdominal wall, omentum, and pelvis after enucleation of hysteromyoma treated in our hospital.

## Case Presentation

### Chief Complaint

A 37-year-old female was admitted to our hospital for palpable masses in the lower abdominal wall for 2 years. The masses had grown rapidly in recent half-year, and the bigger was about 3 cm in diameter.

### History of Present Illness

The patient had touched the active masses located in the lower end of the incision on the lower abdominal wall, with a diameter of about 1 cm and no tenderness since 2 years ago. The masses consciously increased before the menstrual period and narrowed after the menstrual period. The patient felt the masses had grown rapidly in recent half a year and the bigger was about 3 cm in diameter. She had a regular menstrual cycle and no previous history of oral contraceptives and other hormone drugs, without abdominal pain, abnormal vaginal bleeding, frequent urination, anal distension or other discomfort.

### History of Past Illness

The patient had a history of laparotomy and myomectomy about 10 years ago, without the use of a power morcellator. It was finally confirmed as uterine myoma, about 8 cm in diameter in the posterior wall of uterine. The histopathology showed the myoma was in rich cell type, with red degeneration. In 2013, the patient underwent one cesarean section in an external hospital and abdominal wall foreign body resection was performed simultaneously. The postoperative pathological was reported multiple leiomyoma with active cell proliferation (under the skin of pubic caruncle, upper segment of the rectus abdominis, and peritoneal incision).

### Personal and Family History

The patient denied the history of diseases such as hypertension, diabetes, and heart disease. Her personal and family history were unremarkable.

### Imaging Examinations

**Figure 1:**
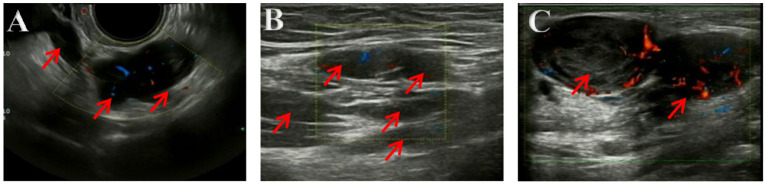
**(A–C)** Ultrasonography imaging. Multiple solid masses in the subcutaneously muscular layer, with size of 2.8 × 2.8 × 1.8 cm in bigger mass. Color Doppler flow imaging (CDFI) showed a strip-like blood flow signal.

**Figure 2:**
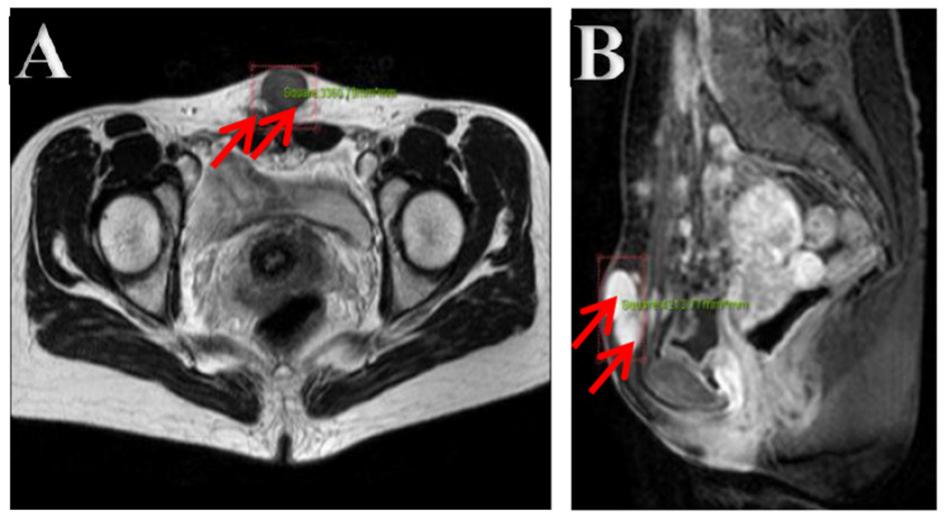
**(A,B)** Pelvic cavity magnetic resonance imaging. Multiple nodes in the abdominal wall, in the right iliac fossa, and behind the uterus, with lower T1- and T2-weighted image. The bigger showed homogeneous enhancement on enhancement images in the soft tissue of the lower abdominal wall.

The ultrasonography showed a 1.5 cm × 1.8 cm × 1.5 cm hypoechoic mass in the posterior wall of the uterus and a 3.8 cm × 2.5 cm × 1.8 cm irregular mass behind the uterus. Color Doppler flow imaging (CDFI) showed a strip-like blood flow signal. In the subcutaneously muscular layer of the lower abdominal wall incision, there also were multiple solid masses with blood flow signal showed (Figures 1A–C). For further diagnosis, the pelvic enhanced MRI was performed, which showed surgical scar shadow in the anterior and lower abdominal wall. T1 and T2 low signal nodules could be seen in the soft tissue around the scar, in the right iliac fossa and behind the uterus. The larger one was located in the soft tissue of the abdominal wall, with a size of 2.6 cm × 1.7 cm × 2.8 cm. The enhanced scan was significantly enhanced but uniform. The size of uterus was normal without abnormal signal shadow found, and the enhancement was uniform (Figures 2A,B). A contrast-computed tomography (CT) scan of the chest also was performed. The texture of both lungs was clear, and the permeability of lung field was good. There was no enlarged lymph node shadow in mediastinum, and there was no abnormality in bilateral pleura and thorax.

### Laboratory Examinations

Laboratory examinations for CA125, CEA, CA724, HE4, CA199 showed no deviation from normal values. Other blood test results were all in the normal spectrums.

### Treatment

**Figure 3:**
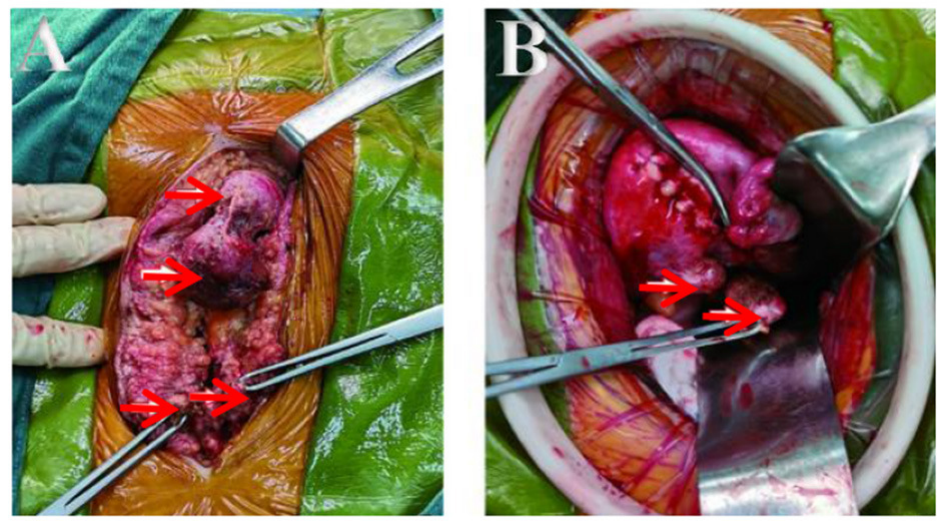
**(A,B)** Lesions seen interoperation. **(A)** Obvious lesions in fat layer and fascia. **(B)** Lesions in the back wall and sacral ligament of the uterus.

The patient underwent an exploratory laparotomy. We noted multiple solid nodules scattered located in the fat layer, fascial layer, intramuscular tissue, and peritoneal layer along the incision, varying in size from 0.5 to 4 cm. After entering into the abdominal cavity, there were extensive pelvic adhesion and multiple nodules found on the edge of greater omentum (3 cm in size), in the posterior wall of the uterus (3 cm in size), and in the uterorectal fossa (4 cm in size) close to rectum. Others were found in the left-posterior wall of uterine (2 cm in size), and on the surface of the right sacral ligament (2 cm in size), which were close to the ureter and surrounding tissue (Figures 3A,B). The activity of the uterus was poor, but both adnexa were normal. The intraoperative frozen section reported the nodules as leiomyoma. Finally, all the visible lesions and partial greater omentum were removed. Accurate hemostasis was performed and no drainage tube was placed to prevent further spread of neoplastic cells to the peritoneal cavity. The patient was given 6 cycles (per 28 days) of gonadotropin releasing hormone analog (GnRHa) to avoid a recurrence. Clinical and radiological follow-ups were strongly recommended for the patient.

### Pathological Examinations

**Figure 4:**
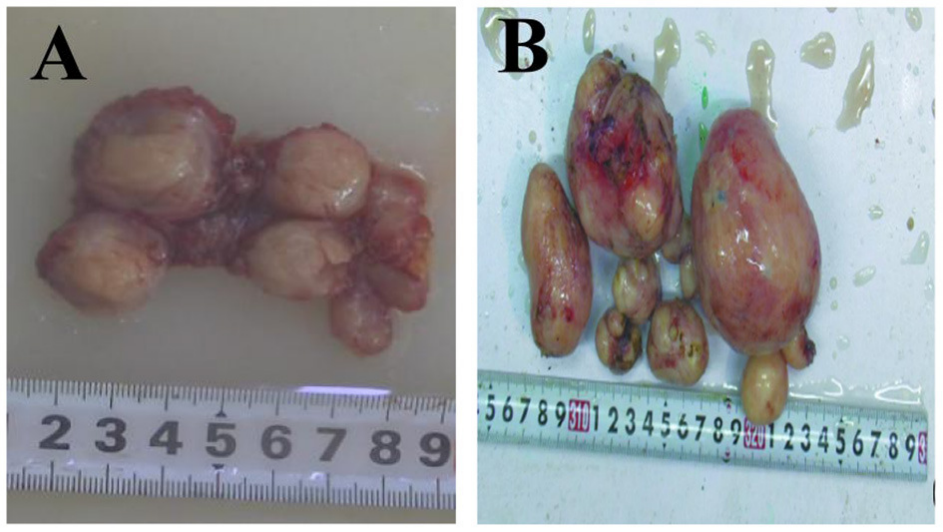
**(A,B)** Grossly tissue. Multiple solid nodules, varying in size from 0.5 to 4 cm.

**Figure 5:**
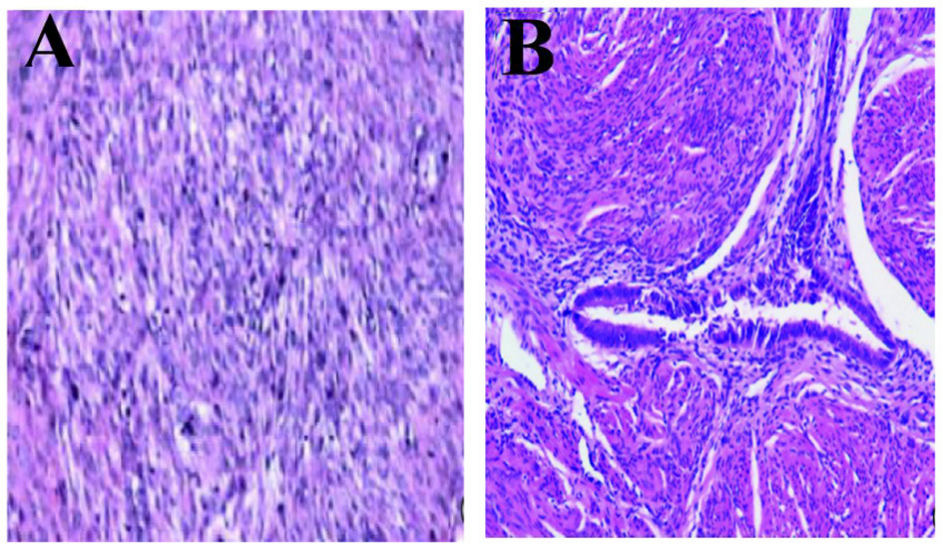
Microscopic observation. **(A)** Pathology microscopically seen for the first surgery, in rich cell type with red degeneration and **(B)** Immunohistochemical finding: SMA(+), Desmin (+), myogenin(–), Ki-67(+2%), HMB-45(–), CD34(–), CD117(–), Dog1(–), ER(–), PR(2+) (H&E, 100×).

The macroscopic tissue showed multiple solid nodules (Figures 4A,B). In microscopic observation, the nodules consisted of arranged spindle cells, with occasional mitotic figures and mild atypia, but no necrosis. Immunohistochemistry showed that SMA(+), Desmin(+), myogenin(–), Ki-67(+2%), HMB-45(–), CD34 (blood S-100) (–), CD117 (–), dog1(–), ER(–), PR(+) (Figures 5A,B).

### Final Diagnosis

The final diagnosis was a BML of abdominal wall and pelvic cavity, in rich cell type.

### Outcome and Follow-Up

The patient supported and cooperated with our treatment very much. Up to now, there is no sign of recurrence or new lesions found by imaging examinations. The patient has no conscious discomfort, and feels so grateful for our operation.

## Discussion

### Etiology and Pathogenesis

Up to now, the etiology and pathogenesis of BML are not clear. Among the proposed hypotheses, lymphovascular spread theory is most commonly accepted, which thinks the tumor cells can be spread along the blood or lymph vessels during the operation. Iatrogenic peritoneal seeding theory from ruptured leiomyoma in myomectomy or hysterectomy is more reasonable to explain the incidence of pelvic BML (6, 7), just as the case reported by us here. It has been suggested that BML may evolve from multi-center proliferation of smooth muscle induced by hormonal stimulation, based on the positive immunohistochemical staining for ER and PR in the tumor cells. The antihormonal therapy can also favor this theory (8). There were some studies have presented cytogenetic mutations in BML, such as 19q, 22q, and 1p terminal deletion, 6p21 and 12q15 rearrangements, involving the HMGA2 and BMP8B (9–11). The mutation of MED12 gene on the long arm of X chromosome(Xq12.1), which is related to the regulation of RNA polymerase II transcription, may also be related to BML (12). Besides, coelomic metaplasia and metastasis of low-grade uterine leiomyosarcoma theories have also been suggested to explain the pathogenesis of BML.

### Diagnosis and Differential Diagnosis

The symptoms of BML vary with the locations of metastasis, and most of them have no obvious clinical manifestations, which can be found during physical examination. When the lung is affected, patients may have cough, chest pain, hemoptysis, hemothorax, pneumothorax, and even dyspnea (13). The patient can feel palpable masses when skin is involved. The lesion located in the spine is rarely reported, which can lead to pain, weakness, or paresthesia (3, 14).

Imaging examination is also mostly non-specific, and it is very easy to be misdiagnosed and missed before operation. Chest computed tomography (CT) is commonly used for auxiliary diagnosis of pulmonary BML. For the suspicious lesions located in the pelvic cavity, abdominal cavity, and other extrapulmonary sites, ultrasonography or MRI can be useful (15). A few reports have indicated that ^18^F-FDG PET-CT is helpful for distinguishing BML from some confused malignant tumors, which show strong or high ^18^F-FDG uptake on PET (16, 17).

Pathology and immunohistochemistry are the gold standard for diagnosis. According to the literature, the histopathological morphology of the tumor at the metastatic site is similar to that of the primary tumor of the uterus. Under the microscope, the tumor cells are composed of bundle or palisade spindle smooth cells, with mild cell morphology, braided arrangement, no obvious nuclear atypia and mitosis, and rare necrotic tissue (18, 19). Immunohistochemical results show that the positive markers of smooth muscle (SMA, desmin, and calponin, etc.) supported the origin of smooth muscle. Combined with the positive staining for ER or PR, it is consistent with the characteristics of hormone-dependent tumors (20). Negativity for S-100, CD-117, DOG-1, CD-34 is useful to exclude neurogenic tumor and gastrointestinal stromal tumors (GISTs). The low proliferative state and low Ki-67 index (<1%) are also helpful to exclude malignant tumor (21).

The lung is the organ most frequently affected by BML, and there need differential diagnosis among PBML and other similar diseases occurring in the lung, such as pulmonary lymphangioleiomyomatosis (LAM) (22), solitary pulmonary fibroma, metastatic uterine leiomyosarcoma (MULMS) (23), and fibroleiomyoma hamartoma. For the pulmonary BML, just as this case introduced here, the differential diagnosis involving pelvic and abdominal cavity is as follows: (1) Leiomyomatosis peritonealis disseminata (LPD): the disease occurs mostly in the peritoneum and intraperitoneal cavity. Multiple myoma-like nodules of different sizes are diffusely distributed, and the texture of the nodules is relatively hard with clear boundary, mostly on the surface of organs, not easy to infiltrate and grow. Immunohistochemistry can be helpful for differential diagnosis (24, 25); (2) Parasitic leiomyoma: it is mostly from the pedicled subserosal leiomyoma which shed its pedicle and continue to grow ectopic, forming a parasitic leiomyoma; (3) Uterine sarcoma: under the microscope, many cells have significant atypia and mitotic activity, often accompanied by coagulation and necrosis; (4) Leiomyoblastoma: it is currently known as epithelioid leiomyoma, which shows characteristics similarity to leiomyosarcoma, with unclear boundary and focal bleeding or necrotic areas. Microscopically, it consists of round or polygonal cell groups with eosinophilic or transparent cytoplasm, mainly such as cell lysosomes, glycogen aggregation, and α SMA myofilament. The cells are negative for desmin and h-caldesmon, while positive for keratin and histone deacetylase 8 (26). (5) Peritoneal carcinoma: the cancerous nodules are usually soft in texture, with invasive growth and diffuse distribution in the pelvis and peritoneum. Most patients are accompanied by a large amount of ascites. Immunohistochemistry shows that cytokeratin S-100 and CD34 associated with malignant transformation or metastasis are positive; (6) Low-grade endometrium stromal sarcoma: the tumor cells are similar to endometrial stromal cells in proliferative stage, with abundant thin-walled spiral arterioles. Immunohistochemistry shows positive for CD10, but negative for myogenic markers, which could be helpful for differential diagnosis. Besides, the spine BML should be distinguished from Schwannoma (15).

### Treatment

Due to the rarity of this entity, there is no unified treatment standard for the disease at home and abroad. For patients with surgical indications, such as single or localized lesions, surgical treatment is still the first choice (27), and close follow-up and endocrine therapy should be supplemented after operation; for young women who cannot accept surgery or have fertility requirements, biopsy should be performed on at least one metastatic lesion to determine the pathological type and source, and then corresponding conservative treatment should be taken. Since the lesions are usually ER and PR positive, suppression of hormone production is the main approach to avoid disease progression or recurrence (28), such as oophorectomy, removal of exogenous estrogen, and pregnancy.

If surgery is accepted, the specific surgical methods should be individually executed. For the postmenopausal patient with pulmonary nodules or hysteromyoma found at the same time, it is a feasible to accept hysterectomy and bilateral salpingo-oophorectomy with biopsy of lung lesion. For the young premenopausal women, only surgical castration by bilateral oophorectomy is an alternative therapy. However, studies have reported that prophylactic resection or diagnostic resection are not recommended (29).

Surgery cannot completely change the pathophysiological basis of BML. Combined drug treatment should be paid attention to prevent and reduce recurrence. For patients who are not suitable or refuse surgical treatment, drug treatment options can be selected, such as long-acting GnRHa and new oral small molecule gonadotropin releasing hormone (GnRH) receptor antagonist. The latter blocks the GnRH signal pathway by competitively binding GnRH receptor in pituitary reversibly reduces the secretion of ovarian sex hormone, estradiol (E2), and progesterone (30) to reduce the occurrence and development of BML. Non-peptide oral GnRH receptor antagonists avoid the down regulation process of 1–2 weeks (31) and take effect quickly. After stopping the drug, they can restore the inhibitory effect on gonadotropin in 24 h and resume the menstrual cycle in about 22 days. Compared with the traditional GnRHa, it has high oral bioavailability, convenient to apply, can avoid the pain of subcutaneous injection, increases the compliance of patients, and can reduce the allergic reaction easily caused by the polypeptide structure in the traditional preparation (32). In addition, it can significantly reduce the symptoms of severe menstrual bleeding in women with hysteromyoma (33).

In this case introduced by us, the metastasis sites were located in the abdominal wall, greater omentum and pelvic cavity, which were rarely reported. Given the age and wishes of the patient, the surgeons only excised the visible lesions as completely as possible, without hysterectomy or bilateral ovaries. But considering its history of recurrence and multiple operations, it is recommended to supplement GnRHa treatment after operation, and should be followed up for a long time. The possibility of new lesions in other systems cannot be ignored.

## Conclusion

To date, there are no standardized guideline-based treatments for BML. However, patients usually can have a favorable prognosis to accept a comprehensive treatment, based on the indolent nature of BML (34). More importantly, long-term surveillance of sex hormone level and imaging follow-ups are required to monitor disease progression (35). For the exploration of the etiology, diagnosis, and treatment of BML, a large number of clinical data still need to be statistically analyzed.

## Data Availability Statement

The original contributions presented in the study are included in the article/supplementary material, further inquiries can be directed to the corresponding author/s.

## Ethics Statement

Written informed consent was obtained from the individual(s) for the publication of any potentially identifiable images or data included in this article.

## Author Contributions

YL and KL were involved in the case design. YL completed the manuscript. TX and MW performed editing pictures. LJ, QL, and KL participated in improving the manuscript. All authors read and approved the final manuscript.

## Funding

This work was supported by the China Medical University and the data permitted by the patient.

## Conflict of Interest

The authors declare that the research was conducted in the absence of any commercial or financial relationships that could be construed as a potential conflict of interest.

## Publisher's Note

All claims expressed in this article are solely those of the authors and do not necessarily represent those of their affiliated organizations, or those of the publisher, the editors and the reviewers. Any product that may be evaluated in this article, or claim that may be made by its manufacturer, is not guaranteed or endorsed by the publisher.
